# Effect of protocol-based family visitation on physiological indicators in ICU patients: a randomized controlled trial

**DOI:** 10.1186/s12871-023-02396-3

**Published:** 2024-01-09

**Authors:** Zahra Nazari-Ostad, Mohammad Namazinia, Fatemeh Hajiabadi, Nahid Aghebati, Habibollah Esmaily, Arash Peivandi Yazdi

**Affiliations:** 1 Department of Medical- Surgical Nursing (MSC Student), School of Nursing and Midwifery, Mashhad University of Medical Sciences, Mashhad, Iran; 2 Department of Nursing, School of Nursing and Midwifery, Torbat Heydariyeh University of Medical Sciences, Torbat Heydariyeh, Iran; 3 Department of Medical- Surgical Nursing, School of Nursing and Midwifery, Mashhad University of Medical Sciences, Mashhad, Iran; 4 Nursing and Midwifery Care Research Center, Mashhad University of Medical Sciences, Mashhad, Iran; 5 Lung Diseases Research Center, Mashhad University of Medical Sciences, Mashhad, Iran; 6 Social Determinants of Health Research Center, Mashhad University of Medical Sciences, Mashhad, Iran; 7 Department of Biostatistics, School of Health, Mashhad University of Medical Sciences, Mashhad, Iran

**Keywords:** Intensive care units, Patient visitation, Protocol-based intervention, Vital signs, Physiological monitoring, Randomized Controlled Trial

## Abstract

**Background:**

Intensive care unit (ICU) patients often experience significant physiological stress. This study evaluated the effect of a defined family visitation protocol on physiological responses in the ICU.

**Methods:**

A randomized, block-randomized clinical trial was conducted on 78 ICU patients at Imam Reza Hospital between February 8, 2017, and August 8, 2017. The intervention group received protocol-based visits, and the control group continued with standard visitation. Block randomization was utilized for group assignments. The primary outcome was the measurement of physiological signs using designated monitoring devices. Data were analyzed using SPSS version 22, employing independent t-tests, Mann-Whitney U test, repeated measures analysis, and Friedman’s test.

**Results:**

The results showed no significant differences in systolic blood pressure, diastolic blood pressure, mean arterial pressure, respiratory rate, and arterial blood oxygen levels between the two groups. However, heart rate in the intervention group was significantly lower in three stages before, during, and after the meaningful visiting (*P* = 0.008).

**Conclusion:**

Protocol-based scheduled family visits in the ICU may reduce physiological stress, as evidenced by a decrease in patients’ heart rate. Implementing tailored visitation protocols sensitive to patient preferences and clinical contexts is advisable, suggesting the integration of family visits into standard care practices for enhanced patient outcomes.

**Trial Registration:**

IRCT20161229031654N2; 25/01/2018; Iranian Registry of Clinical Trials (https://en.irct.ir).

## Introduction

When a patient is hospitalized in the intensive care unit (ICU), it is often due to a life-threatening illness [1]. Despite the presence of advanced equipment and technology in this department aimed at providing better care to patients, some clinical procedures in the ICU can lead to various physical and psychological discomforts for patients [2, 3].

Patients admitted to the ICU encounter significant stressors, such as continuous artificial lighting, constant sounds from monitoring devices and mechanical ventilators, and the absence of meaningful sensory stimuli like touch, pain, and physical discomfort caused by illness [4]. The physiological response of the body to these stressors includes increased metabolic rate, elevated body temperature, increased perspiration, enhanced cardiac contractility, elevated blood pressure, increased heart rate, sodium retention, bronchial dilation, increased respiratory rate, and overall changes in vital signs [5].

Vital signs provide informative data that can be used to identify the patient’s baseline health status. Changes in vital signs can indicate alterations in physiological function, allowing for immediate response and treatment of acute problems [6, 7]. It is estimated that 30 to 70% of patients in the intensive care unit experience severe physiological stress [8]. Temperature, blood pressure, pulse rate, and respiratory rate are the most commonly measured indicators by healthcare providers, reflecting the normal functioning of bodily systems such as circulatory, respiratory, nervous, and endocrine systems [9, 10].

The management of vital signs in specialized care units is based on a combination of pharmacological and non-pharmacological interventions. Evidence suggests that non-pharmacological measures, such as personal contact and verbal communication with family members and their presence, can reduce physiological disturbances in patients admitted to the ICU [11–13].

Families serve as the cornerstone of an individual’s social support network [14–18]. They can provide comfort and reassurance to patients [19–21] and play a vital role in the recovery of patients hospitalized in the intensive care unit [19, 22, 23].

Among these considerations, visiting hours provide an opportunity to involve family members, especially in cases where patient visitation is restricted or prohibited in the ICU [24]. Visitation aids patients in coping with challenging conditions [25, 26]. Despite the importance of visitation for ICU patients, most hospitals currently impose restrictions on visitation [27]. In nursing and medical teams in intensive care units, there is a deep-seated belief that openness and the presence of family members and loved ones are detrimental [28, 29]. However, recent studies have shown beneficial effects alongside the negative ones of family presence [30, 31]. Some sources advocate for entirely open visitation, while others suggest limitations [32]. In a study, scheduled visits were able to improve patient satisfaction with family members in intensive care units [33]. They proposed the need for planned visits, including visits with specific individuals, a lack of desire to visit certain people, and the need for visits at specific times [34].

The necessity of our study arises from the ongoing debates and the inconsistent findings reported in the literature regarding the impact of family visitations on the physiological indicators of patients in intensive care units (ICUs). While previous studies have acknowledged the potential of non-pharmacological measures to mitigate physiological disturbances, the specific outcomes related to protocol-based scheduled family visits remain less understood, with evidence showing both beneficial and adverse effects [30–32].

The current body of literature demonstrates a crucial gap: the need to systematically assess the influence of controlled family visitation protocols on a wide range of physiological indicators such as blood pressure, pulse rate, O2 saturation, and respiratory rate. Studies conducted to date have varied in their methodologies, population, and ICU environments, leading to inconclusive or contradictory results [33–35]. This variability underscores an urgent need to explore this area with a robust and rigorous trial design that can offer clearer insights and potentially reconcile these discrepancies.

The intricate interplay between psychological wellbeing and physiological health forms the cornerstone of our study’s rationale. It is well-documented that psychological interventions can manifest in measurable physiological changes, as the link between a patient’s mental state and physical condition is bidirectional and profound [36, 37].

To address this, the hypothesis has been formulated for investigation in our study “Protocol-based scheduled visiting of family members has a positive impact on physiological indicators such as blood pressure, pulse rate, O2 sat, and respiratory rate of patients admitted to the intensive care unit (ICU).”

To date, the impact of utilizing such scheduled visits on the physiological indicators of patients in the intensive care unit (ICU) has not been investigated. Therefore, the present study aimed to determine the effect of protocol-based visiting on the physiological indicators of patients hospitalized in the ICU.

## Methods

### Trial design

This study employed a randomized two-group clinical trial conducted on 78 hospitalized patients in the Intensive Care Unit (ICU) of Imam Reza Hospital at Mashhad University of Medical Sciences from February 8, 2017, to August 8, 2017.

### Participants

Inclusion criteria for this study encompassed participants aged 18 to 70, stable hemodynamics, no prior history of recognized psychiatric illness, and a Glasgow Coma Scale (GCS) score between 11 and 15. Exclusion criteria included unwillingness to continue participating in the study, non-cooperation of patients’ families, and patient deterioration.

### Intervention

In the intervention group, visits were conducted according to a predefined protocol, with three sessions per day: morning, regular afternoon, and one session at night. In this protocol, the patient’s preferences for specific individuals were determined through an initial assessment of the patient and family members. Visiting were held with the individuals the patient wished to meet, and respect was shown to those whom the patient did not want to meet. Explanations were provided regarding the temporary nature of the study period, ensuring their consent to participate, and conveying necessary information about the patient’s condition. A designated individual received 20-minute individual training sessions on how to conduct visits. This training encompassed proper communication with the patient, bringing items or objects that would bring joy to the patient without violating hospital regulations, maintaining emotional control when interacting with the patient and observing their condition, talking about pleasant memories, individuals, and events that were pleasing to the patient. Additionally, they were advised to bring recorded voices of people the patient wished to meet but could not for any reason.

In the control group, visits were conducted using the routine method typically employed in specialized care units, with visits occurring once in the afternoon.

### Outcomes

To collect data, a personal information questionnaire and monitoring equipment were used to measure physiological indicators. The personal information questionnaire included several questions about age, gender, marital status, education, hospitalization history, income status, and connection to mechanical ventilation equipment.

Monitoring equipment, specifically the Saadat Alborz B5 model, installed above each specialized care unit bed, was used to measure physiological indicators. Parameters such as systolic blood pressure, diastolic blood pressure, and mean arterial pressure were measured non-invasively in a semi-upright position with the monitor placed on the right index finger. Oxygen arterial blood saturation was measured using a probe placed on the patient’s right index finger.

Validity of monitoring: The monitoring device’s validity was confirmed due to its calibration and repeated accuracy control, and it is considered a reliable tool for measuring patients’ physiological indicators.

### Sample size and randomization

Sample size calculation, based on a comparison of means with a 95% confidence interval and an 80% test power, estimated 35 individuals in each group.

Participants meeting the inclusion criteria were assigned to either the intervention or control group using a block randomization technique. This was facilitated by the website randomization.org. Blocks were configured to ensure an equal number of participants in each group at any given time. The sequence was concealed until the interventions were assigned to avoid selection bias.

Upon enrollment, the first participant was randomly assigned to the intervention group. Following this, subsequent participants were allocated, maintaining an equal number per block. This process was sequential: New participants were not assigned to the control group until the entire intervention group’s block of participants was discharged or completed the study intervention. Once a block for the intervention group was completed, the same process was initiated for the control group—no new participants were included in the intervention group until the control block was complete.

This method ensured that the assignment of participants to the intervention or control groups was not predictable and preserved the integrity of the randomization process throughout the study. Furthermore, to minimize bias, blinding was implemented at the level of outcome assessors and statisticians. These individuals were kept unaware of group assignments to ensure the impartiality of data collection and analysis.

## Statistical methods

To describe quantitative data, measures such as means and standard deviations were used, while for variables with non-normal distributions, median and interquartile range were utilized. Qualitative data were described using frequency distribution tables or diagrams. We used the Shapiro-Wilk test for normality. In data analysis, independent t-tests were employed to compare quantitative variables between the intervention and control groups, while chi-square tests were used for qualitative variables. Non-parametric tests like the Mann-Whitney U test were used when quantitative variables were not normally distributed. Within-group analysis of variables was conducted using the Friedman and repeated measures analysis of variance tests. Data were analyzed using SPSS version 22, and a significance level of 0.05 was considered. In light of the observed gender differences between our intervention and control groups (*p* = 0.024), we have now included gender as a covariate in our analyses. This adjustment ensures that the comparisons of physiological indicators between the two groups are not confounded by this variable. All results reported henceforth reflect the inclusion of gender as a covariate.

## Results

**Fig. 1:**
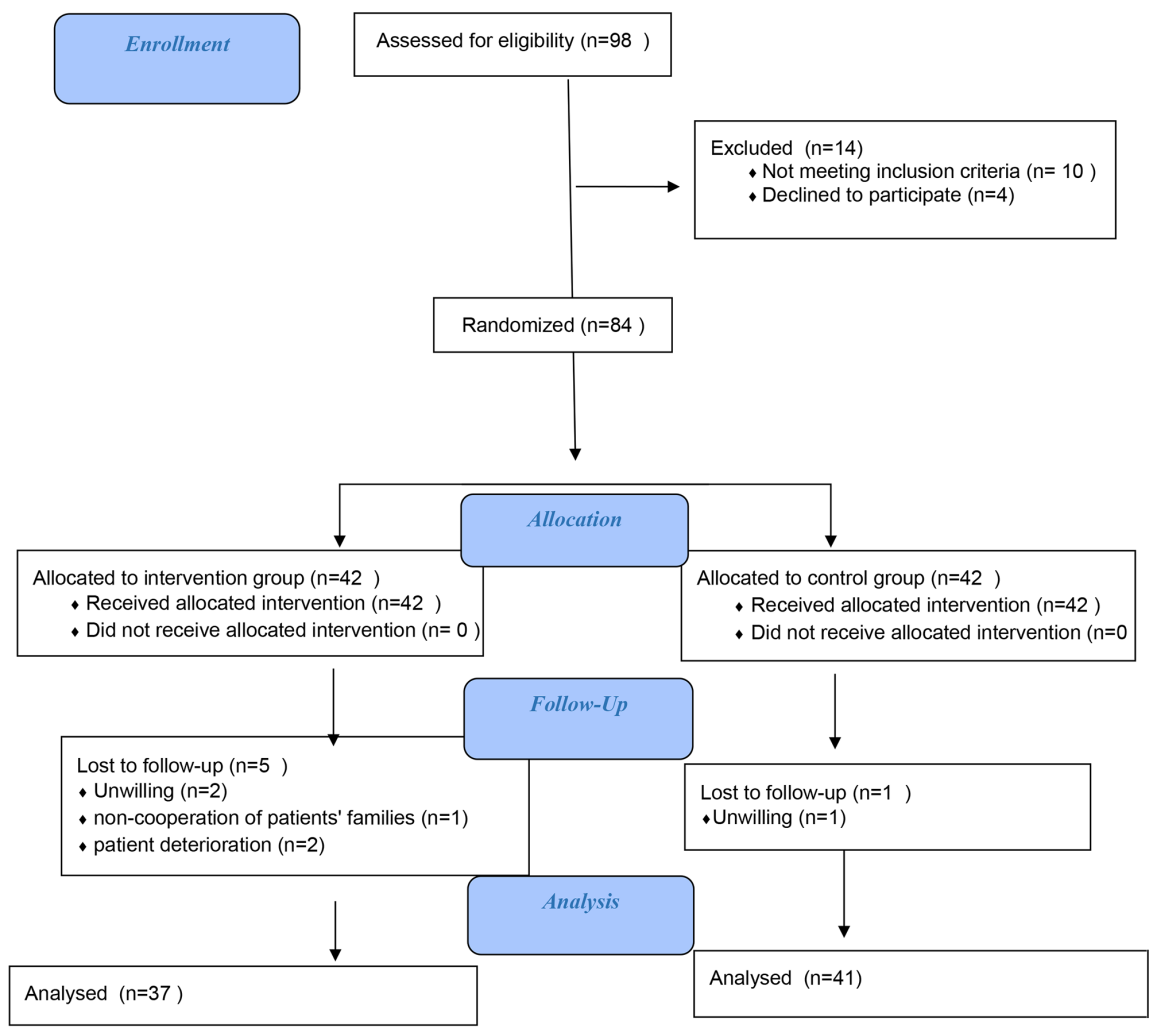
CONSORT Flow Chart of participants

To ensure greater confidence and facilitate subgroup analysis, accounting for a 20% dropout rate, 42 individuals were included in each group. In total, 78 individuals remained in the study after excluding 5 individuals from the intervention group and 1 individual from the control group (Fig. 1).

In assessing the demographic and clinical characteristics of the study population, we observed that the intervention group was comprised of 14 male individuals (37.8%), while the control group contained 26 male individuals (63.4%). In terms of marital status, the majority in both the intervention group (27 individuals or 73.0%) and in the control group (32 individuals or 78.0%) were married. Statistical analyses did not reveal significant differences between the two groups in terms of age, education, marital status, income status, hospitalization history, and connection to mechanical ventilation equipment, with all *p*-values greater than 0.05. These findings suggest that the two groups were well-matched and comparable across these demographic and clinical variables (Table 1).

**Table 1:** Demographic variables of the Intervention and Control groups

Variable		Group		*P* value
		Intervention	Control	
Age (mean ± SD)		42.2 ± 9.8	45.1 ± 3.7	***P = 0.429
Sex n (%)	Male	14 (37.8)	26 (63.4)	****P = 0.024
	Female	23 (62.2)	15 (36.5)	
Marital Status n (%)	Single	4 (10.8)	6 (14.6)	****P = 0.523
	Married	27 (73.0)	32 (78.0)	
	Deceased Wife	6 (16.2)	3 (7.3)	
Education n (%)	Elementary	7 (18.9)	12 (29.3)	***P = 0.121
	Middle School	9 (24.3)	15 (30.8)	
	Diploma	17 (45.9)	9 (33.3)	
	College Education	4 (10.8)	5 (11.5)	
History of hospitalization in n (%)	Yes	8 (21.6)	10 (24.4)	****P = 0.772
	No	29 (78.4)	31 (75.6)	
Income Level n (%)	Less than enough	18 (48.6)	26 (63.4)	***P = 0.177
	Enough	17 (45.9)	14 (39.7)	
	More than enough	2 (5.4)	1 (3.8)	
Under mechanical ventilation n (%)	Yes	8 (21.6)	9 (22.0)	****P = 0.972
	No	29 (78.4)	32 (78.0)	

**** Chi-square *** Mann-Whitney

However, the distribution of gender differed significantly between the groups, as determined by the chi-square test (*p* = 0.024), indicating a higher proportion of males in the control group. This difference has been controlled for in subsequent analyses to ensure that any observed effects on physiological outcomes are not confounded by gender distribution.

Focusing on the primary outcomes of the study — physiological indicators such as systolic blood pressure (SBP), diastolic blood pressure (DBP), arterial oxygen saturation (O2sat), mean arterial pressure (MAP), heart rate (HR), and respiratory rate (RR) — we observed the following:

Prior to the intervention (pre-visiting phase), there were no statistically significant differences between the intervention and control group for SBP, DBP, O2sat, and MAP values, with *p*-values exceeding the 0.05 threshold. This suggests that the intervention did not impact these specific physiological indicators.

Differences in HR and RR, however, were found to be statistically significant between the intervention and control groups during the pre-visiting phase (*p* < 0.05), indicating a potential effect of the intervention on these parameters already noticeable at this stage.

During the visitations, the pattern remained consistent for SBP, DBP, HR, O2sat, and MAP with no significant differences detected (*p* > 0.05), reinforcing the absence of immediate effects of family visitations on these indicators.

Conversely, significant differences in RR were observed during the visitation periods between the groups (*p* < 0.05), suggesting the possibility of visitation having an impact on the patients’ respiratory rate.

Of particular note is that within the intervention group, HR significantly reduced across the three time points examined: before, during, and after family visitation (*p* = 0.008). This outcome suggests that protocol-based family visitation may exert a calming effect on patients, as evidenced by a decrease in heart rate. Given the role of HR as an indicator of stress and physiological relaxation, these findings may imply that the presence of family members during visits can positively influence the patient’s emotional and physiologic state.

These results, outlined in Table 2, point to the specific influence of family visitation protocols on certain, but not all, physiological measurements. It is notable that the most significant changes were observed with respect to heart rate, highlighting the potential benefits of protocol-based visitation on this particular physiological stress indicator.

**Table 2:** Mean systolic and diastolic pressures and their standard deviations, mean arterial pressure, respiration rate, heart rate, and and arterial oxygen saturation levels before and after the intervention for participation in both the intervention and control groups

Variable		Group		*P*
		Intervention N(37)	Control N(41)	
		Mean ± SD	Mean ± SD	
SBP	Before The Visit	129.1 ± 3.8	132.1 ± 3.2	*0.477
	During The Visit	131.1 ± 0.8	132.1 ± 2.9	**0.737
	After The Visit	128.1 ± 7.6	129.1 ± 4.7	*0.968
	Difference between before and during the visit	1.8 ± 7.2	-0.6 ± 1.3	**0.268
	Difference between before and after the visit	-0.8 ± 7.4	-3.9 ± 0.2	**0.248
	Difference between after and during the visit	-2.9 ± 4.5	-2.7 ± 9.4	*0.352
Intragroup Comparison		***0.233	****0.182	
DBP	Before The Visit	78.1 ± 8.5	81.9 ± 9.9	*0.080
	During The Visit	80.1 ± 6.1	81.1 ± 3.8	**0.787
	After The Visit	80.1 ± 5.9	80.9 ± 4.7	**0.971
	Difference between before and during the visit	1.6 ± 9.7	-0.5 ± 5.8	**0.093
	Difference between before and after the visit	1.5 ± 7.8	-1.6 ± 5.5	**0.026
	Difference between after and during the visit	-0.7 ± 2.3	-0.6 ± 9.1	**0.605
Intragroup Comparison		****0.160	***0.298	
O2 sat	Before The Visit	95.1 ± 9.6	95.1 ± 4.9	*0.244
	During The Visit	95.1 ± 9.6	95.1 ± 3.9	*0.219
	After The Visit	95.1 ± 7.7	95.1 ± 3.9	**0.295
	Difference between before and during the visit	-0.1 ± 1.0	-0.0 ± 1.6	*0.983
	Difference between before and after the visit	-0.1 ± 2.0	-0.0 ± 1.4	*0.426
	Difference between after and during the visit	-0.0 ± 2.8	-0.0 ± 0.4	*0.397
Intragroup Comparison		****0.125	****0.052	
RR	Before The Visit	22.5 ± 7.2	19.4 ± 2.8	*0.002
	During The Visit	22.5 ± 6.2	19.4 ± 4.6	*0.004
	After The Visit	22.5 ± 5.4	19.4 ± 2.6	**0.005
	Difference between before and during the visit	-0.2 ± 0.8	0.1 ± 3.9	**0.580
	Difference between before and after the visit	-0.3 ± 2.4	0.2 ± 0.7	**0.704
	Difference between after and during the visit	-0.2 ± 2.6	-0.2 ± 2.4	*0.794
Intragroup Comparison		***0.894	****0.981	
HR	Before The Visit	93.1 ± 5.5	84.1 ± 5.4	**0.013
	During The Visit	91.1 ± 4.4	84.1 ± 8.8	**0.075
	After The Visit	90.1 ± 7.6	85.1 ± 0.7	**0.113
	Difference between before and during the visit	-2.5 ± 1.7	0.4 ± 3.8	**0.054
	Difference between before and after the visit	-2.5 ± 7.9	0.5 ± 5.2	**0.012
	Difference between after and during the visit	-0.4 ± 7.4	0.5 ± 3.3	**0.424
Intragroup Comparison		***0.008	***0.817	
MAP	Before The Visit	95.1 ± 8.8	98.1 ± 5.0	**0.315
	During The Visit	97.1 ± 8.4	98.1 ± 5.2	**0.801
	After The Visit	97.1 ± 1.1	96.1 ± 2.4	**0.712
	Difference between before and during the visit	2.6 ± 0.4	-0.5 ± 0.1	*0.242
	Difference between before and after the visit	1.5 ± 3.3	-2.6 ± 3.9	**0.012
	Difference between after and during the visit	-0.6 ± 7.7	-2.6 ± 3.1	**0.279
Intragroup Comparison		***0.135	****0.067	

* Mann-Whitney ** Independent T-test *** Repeated Measures **** Friedman Test

Further details and the statistical significance of other outcomes are presented in Table 2, providing a comprehensive view of the data collected and analyzed in this study (Table 2).

## Discussion

The present study’s findings contribute to a nuanced body of literature with varying conclusions about the physiological impacts of family visits on ICU patients. Although no significant differences were found within our results when considering average systolic blood pressure, diastolic blood pressure, mean arterial pressure, arterial blood oxygen saturation, and respiratory rate between intervention and control groups, a noteworthy decrease in heart rate was observed in the intervention group. This effect was persistent across different stages—before, during, and after visitation—suggesting the potential of family presence to induce a calming effect and reduce cardiovascular stress in ICU patients.

Aligning with this, the study by Jani (2015) indicated that consistent, supportive family caregiving could modulate physiological parameters in patients with spinal cord injuries, hinting at the therapeutic potential of family involvement [13]. Similarly, Rahmani’s study, which determined that structured visitations could lead to a modulation of systolic blood pressure by the third day of hospitalization, supports the theory that the intervention’s timing and consistency are important factors for physiological benefits [38]. In contrast, Akbari et al. (2019) discovered immediate effects, showing a reduction in systolic blood pressure just 30 min after the visit [9], while Salavati et al. (2012) reported no significant changes, prompting considerations of individual patient variability and possible psychophysiological dynamics involved during family interactions [39].

The dynamic nature of diastolic blood pressure responses to family visiting was demonstrated by Mahmoudi et al. (2016), who found that extended visitation times could affect diastolic pressure in a specific direction [35]. Yet, Razaei et al. (2016) experienced an increase in diastolic blood pressure with longer visits, counter to the common presumption of visitation as a universally soothing practice [35]. Kamrani et al. (2010) also reported an increase in diastolic pressure following the start of a visit, reinforcing the notion that patient reactions to family presence are highly individual and may reflect complex emotional and physiological interactions [29].

When examining the impact of family visits on mean arterial pressure, the studies present a multifaceted picture. Kamrani et al. (2010) saw increases after visitations began, contrasting our findings where no significant variation was observed [29]. Salavati et al. (2012) and Basiri Moghadam et al. (2015) also contributed mixed outcomes to this discussion, the former not detecting changes, and the latter observing modulation in stroke patients [39]. These discrepancies underscore the necessity of individualizing visitation protocols to the patient’s needs and conditions for optimal physiological outcomes.

Regarding heart rate, our study mirrors the results of Basiri Moghadam et al. (2015) and Shahvali et al. (2022), where scheduled, structured visits were associated with heart rate reductions, which aligns with the physiological signatures of stress relief and improved patient well-being [40]. This is contrasted, however, with Kamrani et al. (2010), who reported an increase in heart rate, suggesting that visits in certain contexts or without proper structure might inadvertently induce stress [29]. Notably, our study provides a unique perspective by incorporating three visitation sessions with preferred individuals, possibly reinforcing the positive impacts of emotionally supportive visits.

The data on respiratory rate and arterial blood oxygen saturation also deliver mixed insights. While our study and that of Salavati et al. (2012) observed no significant change in respiratory rate, Basiri Moghadam et al. (2015) found it could be positively modified with regular care-focused visitation [39]. The relationships between visitation, therapeutic communications, and minimal restriction policies could offer potential pathways for improving respiratory parameters, which should be further examined [41, 42]. Moreover, the discrepancy in arterial blood oxygen saturation outcomes between studies indicates variable physiological responses that can be attributed to the intervention specifics or patient situational factors [29, 39, 43].

These interpretations emphasize the burgeoning recognition of family visits as an integral component of patient care within specialized care units. While this collective research highlights the delicate balance needed in creating visitation protocols that meet the clinical and emotional needs of patients, it also stresses the importance of customized approaches informed by patient preferences, medical conditions, and individual responses. Ultimately, structured visitation that is well-planned and patient-centered appears to consistently produce beneficial results, advancing patient care and supporting recovery.

In light of the current study findings, the following suggestions are proposed for future research to enhance and broaden the understanding of family visitation protocols in ICU settings:

Patient-Centered Approach: Investigate the efficacy of visitation protocols based on patient preferences and individualized needs, thus promoting patient-centered care in critical settings, Quality of Life Assessments: Incorporate assessments of patient and family-reported outcomes on quality of life and psychological well-being to evaluate the broader impacts of family visitation protocols, Longitudinal Studies: Implement longitudinal research to track the long-term effects of regulated family visitation on ICU patients, assessing whether immediate physiological benefits correspond with improved long-term recovery outcomes, Extended Protocols: Develop and test comprehensive protocols that include additional non-pharmacological interventions—like guided imagery or mindfulness—to complement the visitation sessions, Different ICU Settings: Replicate this study across various hospital environments and cultural contexts to evaluate the generalizability of the findings and to accommodate diverse patient populations, Vigilance in Caregiver Training: Explore diverse training methodologies for caregivers to optimize their interactions with patients during visits, aiming to enhance the effectiveness of visitation protocols, Impact on Healthcare Providers: Research the impact of visitation protocols on the workloads and perspectives of nursing and medical teams within ICU environments, taking into account their standpoint on open versus restricted visiting hours, Technological Augmentation: Study the integration of innovative technologies, such as virtual reality, to simulate the presence of family members, potentially offering alternative means of social support for patients unable to receive visitors, Economic Evaluation: Focus on the economic implications of implementing family visitation protocols, analyzing the correlation between improved physiological indicators and potential reductions in hospitalization costs and length of ICU stays, Visitation Impact on Staff: Understand the effects of family visitation on ICU staff, examining how it influences their workload, stress levels, and job satisfaction with the goal of optimizing visitation policies to improve working conditions.

The limitations of our study are multifaceted and notably reflective of the intricate nature of ICU settings. Achieving caregiver cooperation for the protocol-based visitation intervention posed a significant challenge—addressed through proactive communication and logistical support. The nursing staff’s engagement in implementing the intervention also required significant facilitation. Additionally, the restrictive inclusion criteria, while necessary to ensure patient safety and reliability of results, limited participant heterogeneity and potentially extended the duration needed for adequate sample acquisition. Furthermore, our study’s design within a single internal ICU setting may limit the generalizability of our findings across different ICUs with varied patient profiles and procedural norms. These limitations underpin a call for cautious interpretation of our outcomes and advocate for expansive future research to bolster evidence for the efficacy of protocol-based family visitation in critical care environments.

## Conclusion

protocol-based family visits constitute a non-pharmacological intervention that may help in reducing physiological stress indicators such as heart rate in ICU patients. The findings support the cautious implementation of visitation protocols in intensive care units, tailored to both patient preferences and clinical needs. We recommend incorporating family visitation guidelines into routine care practices while seeking to continually analyze and improve upon these strategies for the optimum benefit of patients.

## Data Availability

The datasets generated in the present study are available from the corresponding author upon reasonable request.

## Abbreviations

ICU: Intensive care unit
